# Soybean oil-based HFD induces gut dysbiosis that leads to steatosis, hepatic inflammation and insulin resistance in mice

**DOI:** 10.3389/fmicb.2024.1407258

**Published:** 2024-08-06

**Authors:** Texy Jacob, Sardar Sindhu, Amal Hasan, Md. Zubbair Malik, Hossein Arefanian, Fatema Al-Rashed, Rasheeba Nizam, Shihab Kochumon, Reeby Thomas, Fatemah Bahman, Steve Shenouda, Ajit Wilson, Nadeem Akther, Areej Al-Roub, Nermeen Abukhalaf, Shaima Albeloushi, Mohamed Abu-Farha, Ashraf Al Madhoun, Fawaz Alzaid, Thangavel Alphonse Thanaraj, Heikki A. Koistinen, Jaakko Tuomilehto, Fahd Al-Mulla, Rasheed Ahmad

**Affiliations:** ^1^Dasman Diabetes Institute, Dasman, Kuwait; ^2^INSERM UMR-S1151, CNRS UMR-S8253, Institut Necker Enfants Malades, Université Paris Cité, Paris, France; ^3^Department of Medicine, University of Helsinki and Helsinki University Hospital, Helsinki, Finland; ^4^Department of Public Health and Welfare, Finnish Institute for Health and Welfare, Helsinki, Finland; ^5^Minerva Foundation Institute for Medical Research, Helsinki, Finland; ^6^Department of Public Health, University of Helsinki, Helsinki, Finland

**Keywords:** gut dysbiosis, sucrose free HFD, steatosis, insulin resistance, inflammation

## Abstract

High-fat diets (HFDs) shape the gut microbiome and promote obesity, inflammation, and liver steatosis. Fish and soybean are part of a healthy diet; however, the impact of these fats, in the absence of sucrose, on gut microbial dysbiosis and its association with liver steatosis remains unclear. Here, we investigated the effect of sucrose-free soybean oil-and fish oil-based high fat diets (HFDs) (SF-Soy-HFD and SF-Fish-HFD, respectively) on gut dysbiosis, obesity, steatosis, hepatic inflammation, and insulin resistance. C57BL/6 mice were fed these HFDs for 24 weeks. Both diets had comparable effects on liver and total body weights. But 16S-rRNA sequencing of the gut content revealed induction of gut dysbiosis at different taxonomic levels. The microbial communities were clearly separated, showing differential dysbiosis between the two HFDs. Compared with the SF-Fish-HFD control group, the SF-Soy-HFD group had an increased abundance of Bacteroidetes, Firmicutes, and Deferribacteres, but a lower abundance of Verrucomicrobia. The Clostridia/Bacteroidia (C/B) ratio was higher in the SF-Soy-HFD group (3.11) than in the SF-Fish-HFD group (2.5). Conversely, the Verrucomicrobiacae/S24_7 (also known as Muribaculaceae family) ratio was lower in the SF-Soy-HFD group (0.02) than that in the SF-Fish-HFD group (0.75). The SF-Soy-HFD group had a positive association with S24_7, Clostridiales, Allobaculum, Coriobacteriaceae, Adlercreutzia, Christensenellaceae, Lactococcus, and Oscillospira, but was related to a lower abundance of Akkermansia, which maintains gut barrier integrity. The gut microbiota in the SF-Soy-HFD group had predicted associations with host genes related to fatty liver and inflammatory pathways. Mice fed the SF-Soy-HFD developed liver steatosis and showed increased transcript levels of genes associated with *de novo* lipogenesis (Acaca, Fasn, Scd1, Elovl6) and cholesterol synthesis (Hmgcr) pathways compared to those in the SF-Fish-HFD-group. No differences were observed in the expression of fat uptake genes (Cd36 and Fabp1). The expression of the fat efflux gene (Mttp) was reduced in the SF-Soy-HFD group. Moreover, hepatic inflammation markers (Tnfa and Il1b) were notably expressed in SF-Soy-HFD-fed mice. In conclusion, SF-Soy-HFD feeding induced gut dysbiosis in mice, leading to steatosis, hepatic inflammation, and impaired glucose homeostasis.

## Graphical abstract

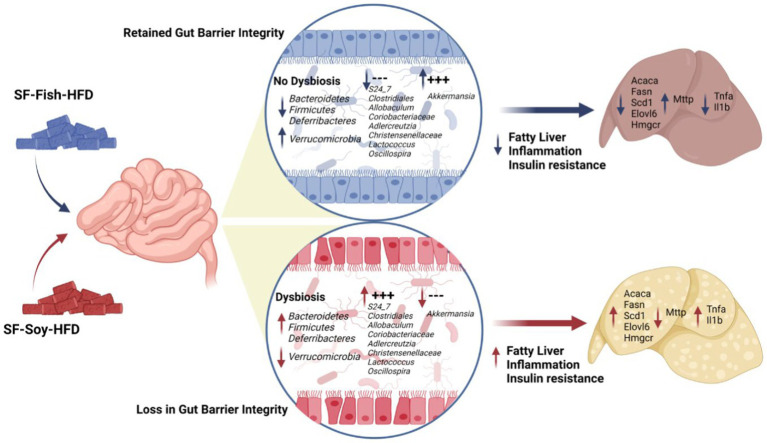

## Introduction

Obesity is a global health problem that predisposes patients to several metabolic disorders such as type 2 diabetes (T2D) and non-alcoholic fatty liver disease (NAFLD). Obesity and associated metabolic disorders are strongly influenced by the gut microbiome. The gut microbiome itself is influenced by a variety of factors, including lifestyle, genetics, drugs, and diet (Weersma et al., 2020; Qin et al., 2022). Dietary fats, in particular, are a valuable source of energy, providing substrates for metabolic processes and short-and long-term energy homeostasis. Dietary fats affect the richness of microbes in the gastrointestinal tract (Lamichhane et al., 2021) as well as modify the structure and functional characteristics of bacteria in the gut (Mokkala et al., 2020; Basak et al., 2022). The gut microbiota breaks down certain food components and contributes to the biosynthesis of metabolic intermediates. Gut bacteria can also interact directly or indirectly with key immune components, including gut-associated lymphoid tissues and phagocytes, to regulate local mucosal homeostasis and maintain the balance between host defense and immune tolerance (Jiao et al., 2020). Given these properties, gut dysbiosis can contribute to several conditions, such as inflammatory bowel disease, obesity, T2D, and fatty liver disease (Gomaa, 2020; Aldars-García et al., 2021; Asadi et al., 2022).

Two common sources of dietary fats that interact with gut bacteria and influence host health are soybean and fish oils. Fish oil is rich in omega-3 fatty acids, eicosapentaenoic acid docosahexaenoic acid, which have anti-inflammatory and cardioprotective health benefits (Watanabe and Tatsuno, 2017; Freitas and Campos, 2019). In regard to dietary effects of omega-3 s, their intake has been shown to positively influence the gut microbiota diversity and enhance the richness of healthy microbiome. Fish oil supports microbiome richness as the anti-inflammatory effects of omega-3 s promote a gut environment which is highly supportive for the growth of a wider spectrum of bacterial species. At the same time, fish oil also improves the evenness or relative abundance of different bacterial species by lowering dominance of certain harmful bacteria such as Proteobacteria and increasing abundance of beneficial bacteria such as Lactobacilli and Bifidobacteria. Thus, fish oil dietary supplementation tends to promote a more balanced gut microbial ecosystem. Fish oil feeding is also associated with an increase in bacterial species producing short-chain fatty acids (SCFAs) which are critical to the maintenance of gut health and microbial richness. In contrast, soybean oil contains more omega-6 fatty acids, which are associated with increased inflammation (Innes and Calder, 2018). Since high intake of omega-6 fatty acids is associated with pro-inflammatory effects (Innes and Calder, 2018), it negatively impacts the bacterial diversity and may induce gut dysbiosis (Selmin et al., 2021). Animal model studies have shown that diets rich in fish oil increase the abundance of beneficial bacteria, such as *Bifidobacterium* and *Lactobacillus* while decreasing the abundance of undesirable bacteria, such as *Clostridium* and *Desulfovibrio* in the gut (Li et al., 2017; Faccinetto-Beltrán et al., 2022). Such changes in the composition of the gut microbiota have been associated with favorable metabolic outcomes, such as decreased inflammation and improved glycemic control (Thomas et al., 2022). Diets rich in soybean oil also affect gut bacteria, reducing the abundance of anti-inflammatory bacteria, such as *Akkermansia* and *Bifidobacterium* and increasing pro-inflammatory bacteria, such as *Bilophila* and *Turicibacter* (Jamar and Pisani, 2022; Sudheer et al., 2022). Notably, the effect of soybean oil on microbial richness can vary, depending on the intake amounts and the host’s health. Soybean oil supplementation often results in decreased microbiome evenness as its omega-6 content supports the growth of certain pathogenic bacteria over beneficial bacteria. Based on these studies, a diet rich in fish oil can be considered beneficial, whereas a diet rich in soybean oil may have detrimental effects on the gut microbiome and metabolic health (Deol et al., 2017). Overall, fish oil-based diets generally promote the bacterial diversity, richness, and evenness due to the anti-inflammatory properties of omega-3 s while soybean oil-based diets may reduce the bacterial diversity, richness, and evenness due to pro-inflammatory effect of omega-6 fatty acids (Ghosh et al., 2013a,b).

NAFLD is a common and potentially serious metabolic complication that is associated with obesity. The liver is particularly sensitive to changes in the gut because it is the first organ/tissue to encounter blood coming from the intestine via the portal vein. Blood is rich in nutrients, bacterial products, and metabolites. Indeed, studies have shown that alterations in the gut microbiome diversity and composition may contribute to the onset and progression of NAFLD (Ni et al., 2020; Henry et al., 2022). Individuals with NAFLD have distinct microbial profiles compared to their healthy counterparts. Regarding the changes in microbiome diversity, patients with NAFLD exhibit reduced microbial richness (number of species) and evenness (relative abundance) or lower alpha diversity, i.e., there are fewer types of colonizing bacteria in the gut and there is an imbalance in the bacterial communities present (Tokuhara, 2021). On the other hand, individuals with NAFLD show significant differences in beta diversity, implying that the gut microbiome composition in NAFLD is distinct from that of healthy individuals (Cai et al., 2024). Regarding NAFLD-associated shifts in bacterial composition, the Firmicutes to Bacteroidetes (F/B) ratio is often increased in patients with NAFLD, which suggests a link between NAFLD and obesity and other metabolic disorders (Vallianou et al., 2021). Numerous studies have attempted to identify the microbial species or pathways associated with NAFLD and its metabolic consequences (Masoodi et al., 2021; Oh et al., 2021). Patients with NAFLD show gut microbiome shift to certain pathogens such as Proteobacteria, especially the members of Enterobacteriaceae family, including *Escherichia coli* (Boursier et al., 2016). On the contrary, there is a decrease in the beneficial bacteria, such as those belonging to genera Lactobacillus and Bifidobacterium (Boursier et al., 2016). In NAFLD, there is also a reduction in SCFAs-producing bacteria, leading to compromised gut permeability and increased inflammation (Rau et al., 2018). Besides, increased numbers of pathobionts in the gut are also associated with NAFLD progression (Hrncir et al., 2021). The commonly identified pathways were related to inflammation, hepatic stress response, and lipid metabolism in NAFLD (Sehgal et al., 2022; Xu et al., 2022; Zhao et al., 2022). Gut bacteria could be implicated in the etiology of NAFLD (Tilg et al., 2020). Bacterial lipopolysaccharides (LPS) and toxic microbial metabolites released during dysbiosis and leaky gut state can induce inflammation and contribute to systemic metabolic dysregulation (Zhang et al., 2021; Xiao et al., 2022). Other potential mechanisms include the altered metabolism of bile acids, carbohydrates, and fatty acids which results in fat accumulation and insulin resistance and the altered immune responses, promoting chronic inflammation and hepatic disease progression.

Given the role gut bacteria play in metabolic diseases and susceptibility to NAFLD, along with the microbiome shifts attributable to different fat sources (soybean, fish), it remains unclear how these dietary fats shape gut microbiota, in the absence of dietary sucrose, to affect the induction of NAFLD in mice. To address this, we examined the gut microbial changes under the influence of soybean oil and fish oil-based high fat diet (HFD) feeding in mice with regard to steatosis, liver inflammation, and systemic glucose homeostasis.

## Materials and methods

### Animals

Animal experimental procedures were performed in accordance with the National Institutes of Health guidelines for the care and use of laboratory animals, and were reviewed and approved by the Institutional Animal Care and Ethics Committee (Approval No. RA AM 2016-007). C57BL/6 mice were purchased from Jackson Laboratory, bred in the Dasman Diabetes Institute’s Animal Core Facility, and fed *ad libitum* on a standard chow diet. Mice were housed in a temperature-controlled room (23°C) and maintained on a 12-h dark/12-h light cycle. All experiments were performed using 8–10 weeks old mice. The mice were randomly divided into two groups, with 5–6 mice per group per cage, and body weight gains were recorded. Two types of sucrose-free HFDs (45% kcal fat) were used i.e.: the soybean oil-based experimental diet (D18060403, Research Diets Inc.) and the fish oil-based diet (D18060407, Research Diets Inc.). Fish oil based HFD cohort is being used as a control for comparison for various studies conducted simultaneously with the various HFDs based on different fat sources. The body weight and food intake of the mice were recorded weekly. At 21–22 weeks of dietary intervention, the oral glucose tolerance test (OGTT) and insulin tolerance test (ITT) were performed. The mice were sacrificed at the end of the dietary intervention (24 weeks), and all tissues and organs were collected and flash-frozen in liquid nitrogen. Blood samples were collected for isolation. Plasma. All tissues and plasma samples were stored at −80°C until use. Liver tissues were fixed in 10% neutral buffered formalin and preserved by paraffin embedding. The fecal content was aseptically collected from each mouse and stored at −80°C until use characteristics of mice fed a soyabean or fish oil-based high-fat diet were presented in Supplementary Table S1.

### Oral glucose tolerance test and insulin tolerance test

To perform the OGTT at 21 weeks of feeding, the mice were fasted for 12 h. Glucose was administered orally to each mouse at a rate of 1 g/kg body weight, and blood glucose levels were measured at different time points at 0, 15, 30, 45, 60, 90, 120, 150, and 180 min (Nagy and Einwallner, 2018).

For ITT at 22 weeks of feeding, the mice were fasted for 4 h, and insulin was administered to each mouse intraperitoneally at a rate of 0.75 U/kg body weight. Blood glucose levels were determined at 0, 15, 30, 45, 60, and 90 min as described (Nagy and Einwallner, 2018).

### Plasma measurements

Plasma metabolic hormone levels, including insulin, C-peptide, glucagon, amylin, leptin, PYY, PP, ghrelin, GLP-1, GIP, and resistin, were measured using a 15-plex kit (Cat. # MMHE-44K, MILLIPLEX Mouse Metabolic Hormone Expanded Panel-Metabolism Multiplex Assay, Millipore, Burlington, MA, United States) according to the manufacturer’s instructions.

### Histological analysis

Immunohistochemistry (IHC) was performed for F4/80 antigen staining. Paraffin-embedded liver tissue sections (4 μm thick) were deparaffinized in xylene and rehydrated using descending grades of ethanol (100, 95, and 75%) in water. Antigen retrieval was carried out using a target retrieval solution (pH 6.0; Dako, Glostrup, Denmark) by boiling in a pressure cooker for 8 min and cooling for 15 min. After PBS washing, endogenous peroxidase activity was blocked with 3% H2O2 for 30 min, and non-specific antibody binding was blocked with 5% nonfat milk (1 h), followed by 1% bovine serum albumin (BSA) solution (1 h). Samples were incubated overnight at room temperature with a primary rabbit polyclonal antibody against F4/80 (1:100 dilution, Abcam^®^ ab100790, pH 6.0, Cambridge, MA, United States). After washing with PBS (0.5% Tween), samples were incubated for 1 h with secondary horseradish peroxidase (HRP)-conjugated goat anti-rabbit antibody (EnVision Kit, Dako, Glostrup, Denmark), and color was developed using chromogenic 3,3′-diaminobenzidine (DAB) substrate. Samples were washed under running tap water, lightly counterstained with Harris hematoxylin, dehydrated using ascending grades of ethanol (75, 95, and 100%), cleared in xylene, and mounted in dibutylphthalate xylene (DPX). Digital photomicrographs of adipose tissue sections [20×; PanoramicScan II, 3DHistech, Hungary. URL: https://www.3dhistech.com/products-and-software/hardware/pannoramic-digital-slide-scanners/pannoramic-scan-2/ (accessed on August 10, 2021)] was used to quantify the staining in 10 different regions and assess the regional heterogeneity in the tissue samples. The regions were outlined using the Aperio ImageScope software [Aperio Vista, CA, United States. URL: https://aperio-imagescope.software.informer.com/9.0/ (accessed August 10, 2021)]. The apio-positive pixel count algorithm (version 9) integrated into Imagescope Software was used to quantify the intensity of specific staining in the region. The number of positive pixels was normalized to the total number of pixels (positive and negative) to account for variations in the size of the sampled region. Color and intensity thresholds were set to detect immunostaining as positive and background as negative pixels. Once set, all slides were analyzed using the same parameters. The resultant color markup for the analysis was confirmed for each slide. Liver tissue samples mounted on slides were also processed for hematoxylin-eosin (H&E) staining and Oil Red O staining for fat content following standard protocols, as described (Fengler et al., 2016; Konstantopoulos et al., 2017).

### Gene expression analysis by qRT-PCR

Total RNA was extracted from the liver tissue using an RNeasy Mini kit (Qiagen, Hilden, Germany). Total RNA (1 μg of total RNA was reverse-transcribed into cDNA using the High Capacity cDNA Reverse Transcription Kit, Cat. #4368814, Thermo Scientific, Baltics UAB North America, Vilmus, Lithuania). Quantitative real-time PCR was performed on a QuantStudio^™^ 5 Real-Time PCR System using TaqMan Master Mix reagents and gene-specific TaqMan assays (Cat. #4369016, Thermo Scientific, Baltics UAB North America, Vilmus, Lithuania). Each reaction was performed in triplicate under standard reaction conditions. The target gene cycle threshold (Ct) values were normalized against GAPDH Ct values, and gene expression levels relative to the control were calculated using the 2^−ΔΔCT^ method. Gene-specific primers used are listed in Supplementary Table S2.

### Microbiome sequencing

DNA was extracted from mouse fecal samples using the Qiamp DNA Fast Stool Mini Kit (Qiagen, Germany) and quantified using a Qubit fluorometer 4 (Thermo Fisher Scientific, Waltham, MA, United States) following the manufacturer’s instructions. A total of 5 ng/μL of microbial DNA was amplified using gene-specific primers that target the bacterial 16S rRNA V3 and V4 regions. The full-length primers with overhang adapter sequences used were as follows:16S Amplicon PCR Forward Primer, 5′-TCGTCGGCAGCGTCAGATGTGTATAAGAGACAGCCTACGGGNGGCWGCAG; 16S Amplicon PCR Reverse Primer, 5′-GTCTCGTGGGCTCGGAGATGTGTATAAGAGACAGGACTACHVGGGTATCTAATCC. PCR was carried out using the KAPA HiFi HotStart Ready Mix PCR mix kit (Roche Diagnostics, Indianapolis, IN, United States) following the manufacturer’s protocol. The resulting PCR product was confirmed on a bioanalyzer using a high-sensitivity DNA chip (Agilent Technologies, Inc., Santa Clara, CA, United States), purified using AMPure XP beads (Agilent Technologies, Inc., Santa Clara, CA, United States), and bound to dual indices using the Nextera XT Index Kit (Illumina Inc., San Diego, CA, United States), following the manufacturer’s recommendations. Purified and normalized libraries were multiplexed for up to 24 samples, and paired-end sequencing was performed using the MiSeq platform (Illumina Inc., San Diego, CA, United States).

### 16S rRNA sequencing data analysis and bioinformatics statistics

The sequence data obtained were analyzed using QIIME2 (Quantitative Insights Into Microbial Ecology) version 2022.8 (Caporaso et al., 2010; Bolyen et al., 2019) and the MicrobiomeAnalyst package (Dhariwal et al., 2017). The forward and reverse reads of the same sample were joined first using QIIME2. The paired reads were demultiplexed and quality-filtered using a Q-score of 25. High-quality amplicon sequence variants (ASVs) were obtained using the Divisive Amplicon Denoising Algorithm-2 (DADA2) algorithm (Ombrello, 2020). Taxonomic profile analysis was performed against the Greengenes2 reference database (DeSantis et al., 2006) and transformed into relative abundance at the phylum, class, order, family, genus, and species levels. After this, Using Greengenes reference database, open-reference operational taxonomic units (OTUs) were picked out from the non-chimeric sequences at 97% similarity. The most abundant read from each OTU was selected as the representative read. Taxonomy associated with the Greengenes database, to which OTUs matched, was assigned to OTUs. To test the two-group differences in the percentage of analyzable read numbers between HFF and HFS, we calculated *p*-values using the Wilcoxon signed-rank test. The OTU table was filtered to remove OTUs containing <10 counts in all samples, and were transformed into relative abundances at the phylum, class, order, family, genus, and species levels.

### Microbiome diversity analysis

Intra-sample (alpha) and inter-sample (beta) diversity analyses were performed as described elsewhere (Lozupone and Knight, 2005; Qin et al., 2012; Yatsunenko et al., 2012). Alpha diversity was calculated using three different measures: Observed, Chao1, Abundance-based coverage estimator (ACE), Shannon index (Shannon, 1997), Simpson index (Nagendra, 2002), and Fisher statistics and phylogenetic diversity (Faith, 1992), performed as the references cited for each. Bacterial genus/spp. data were provided. Beta diversity was calculated using Bray-Curtis, Jaccard index, Jensen-shannon and UniFrac distance matrices to generate two-dimensional Principal Coordinate Analysis (PCoA) plots. Weighted and unweighted UniFrac distance matrices were used to derive the beta diversity of the samples (Lozupone and Knight, 2005) which data could be accessed. Non-parametric Mann–Whitney/Kruskal-Wallis tests were used to determine the statistical significance of α-diversity measures, and permutational MANOVA was used to determine the differences in β-diversity.

### Identification of the biomarker microbiome

Linear discriminant analysis (LDA) effect size (LEfSe) analysis was performed to identify the bacteria for which the relative abundance significantly increased or decreased in each phenotypic category. LEfSe algorithm using the Benjamini-Hochberg false discovery rate (FDR) adjusted *p*-value cutoff of 0.05 and the logarithmic LDA score cutoff of 2.0. LEfSe bar plots were created using the MicrobiomeAnalyst package (Dhariwal et al., 2017). In all analyses, *p*-values were corrected for Benjamini-Hochberg FDR. Random Forest analysis was applied to the 16S rRNA data using machine learning methods developed using the MicrobiomeAnalyst package to identify the most significant microbial features. Features with a minimum prevalence of 10% across samples were included, and those with >0.005 accuracy were considered significant. Data were further transformed to cantered log ratio (CLR) before applying the Random Forest classification algorithm.

### Functional metagenome predictions of gut bacterial communities

Phylogenetic investigation of bacterial communities was performed by reconstructing unobserved states-2 (PICRUSt2) software to predict the microbial content from each gut microbe sample and functionally annotate the data (Langille et al., 2013). The results were further analyzed using the MicrobiomeAnalyst package (Dhariwal et al., 2017). To investigate the metabolic network of the predicted organisms, we used the MetaCyc database (Caspi et al., 2014), which contains data regarding chemical compounds, reactions, enzymes, and metabolic pathways that have been experimentally validated. The correlation between the microbial community and their metabolites was determined using the MicrobiomeAnalyst package (Dhariwal et al., 2017).

### Prediction of biomarker microbiome-epigenome interactions

The human microbiome affects host gene expression because microbiota-secreted proteins, microbiota-derived components, and microbiota-derived metabolites may regulate the host’s physiology by modifying its gene expression. Therefore, biomarker microbiome-epigenome interactions were predicted using the MicrobiomeAnalyst taxon set enrichment analysis (Dhariwal et al., 2017), Human Microbe-Disease Association Database (HMDAD) (Caspi et al., 2014), and the human microbiome affects the host epigenome (MIAOME) database (Ma et al., 2017; Wang et al., 2022).

## Results

### SF-Soy-HFD and SF-Fish-HFD have differential effects on gut microbiome but not on tissue or body weight

**Figure 1:**
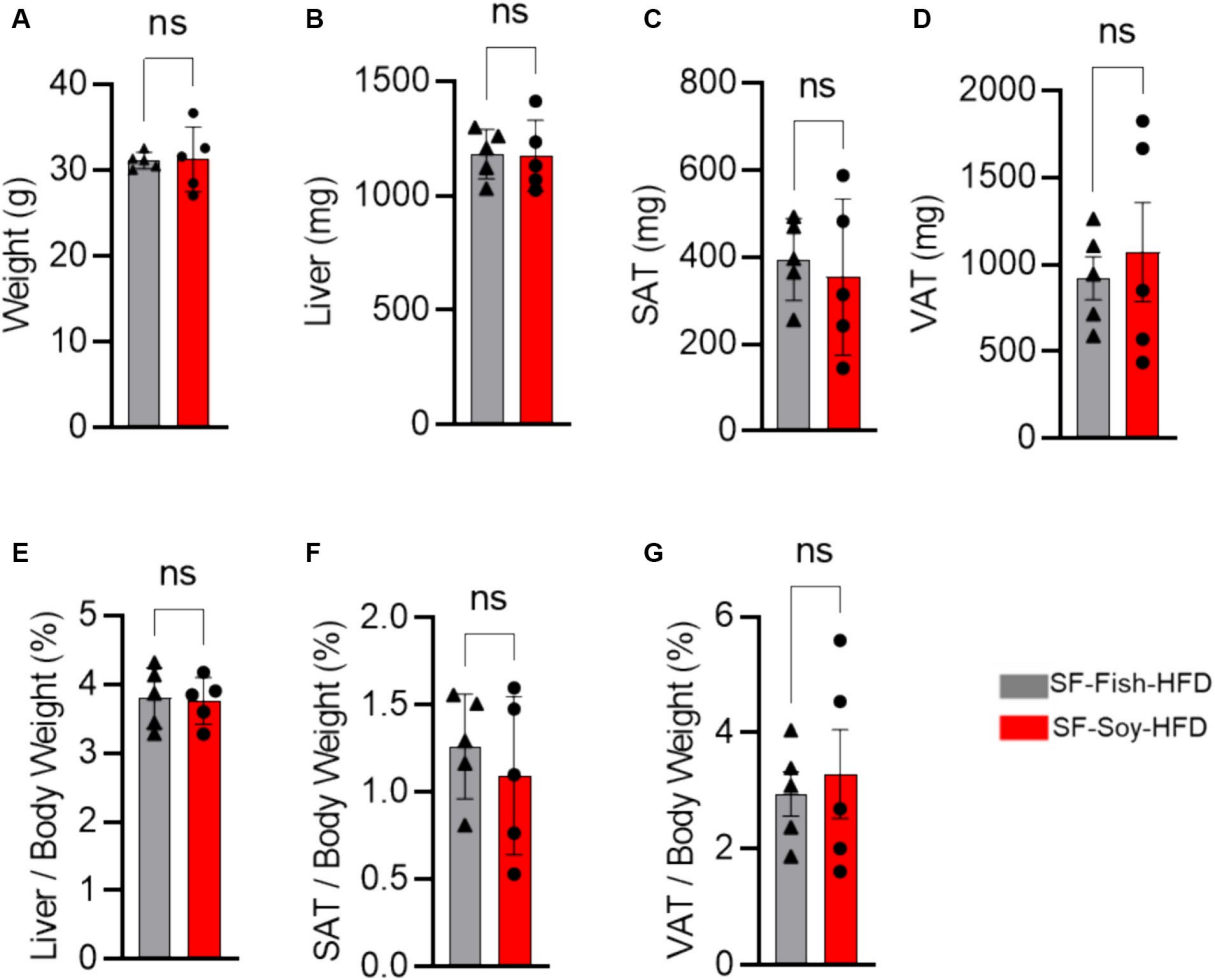
SF-Soy-HFD and SF-Fish-HFD do not have different effects on tissue or total body weight. **(A)** After 24 weeks of dietary intervention of SF-Soy-HFD or SF-Fish-HFD mice body weights were determined. Mice (*n* = 5 of each group) were scarified after 24 weeks of dietary intervention. **(B–D)** Liver, SAT (subcutaneous/inguinal adipose tissue), and VAT (visceral/epididymal adipose tissue) weights of the mice were determined. **(E–G)** Ratios of the liver, SAT or VAT to body weight percentage. Data are shown as means ± SEM. ns, not significant.

We began our investigation by placing mice on the SF-Soy-HFD and SF-Fish HFD regimens. Given the reported benefits of fish oil-based diets (Caesar et al., 2015; Kaliannan et al., 2015), we used the SF-Fish HFD as a control diet for comparison with SF-Soy-HFD. First, we compared end-point body and tissue weights between SF-Soy-HFD and SF-Fish HFD feeding for 24 weeks. To this end, no significant differences in the epididymal (visceral adipose tissue/VAT) and inguinal (subcutaneous adipose tissue/SAT) adipose tissues, liver, or total body weight were observed in mice fed SF-Soy-HFD, compared to those fed SF-Fish-HFD (control group) (Figures 1A–G).

**Figure 2:**
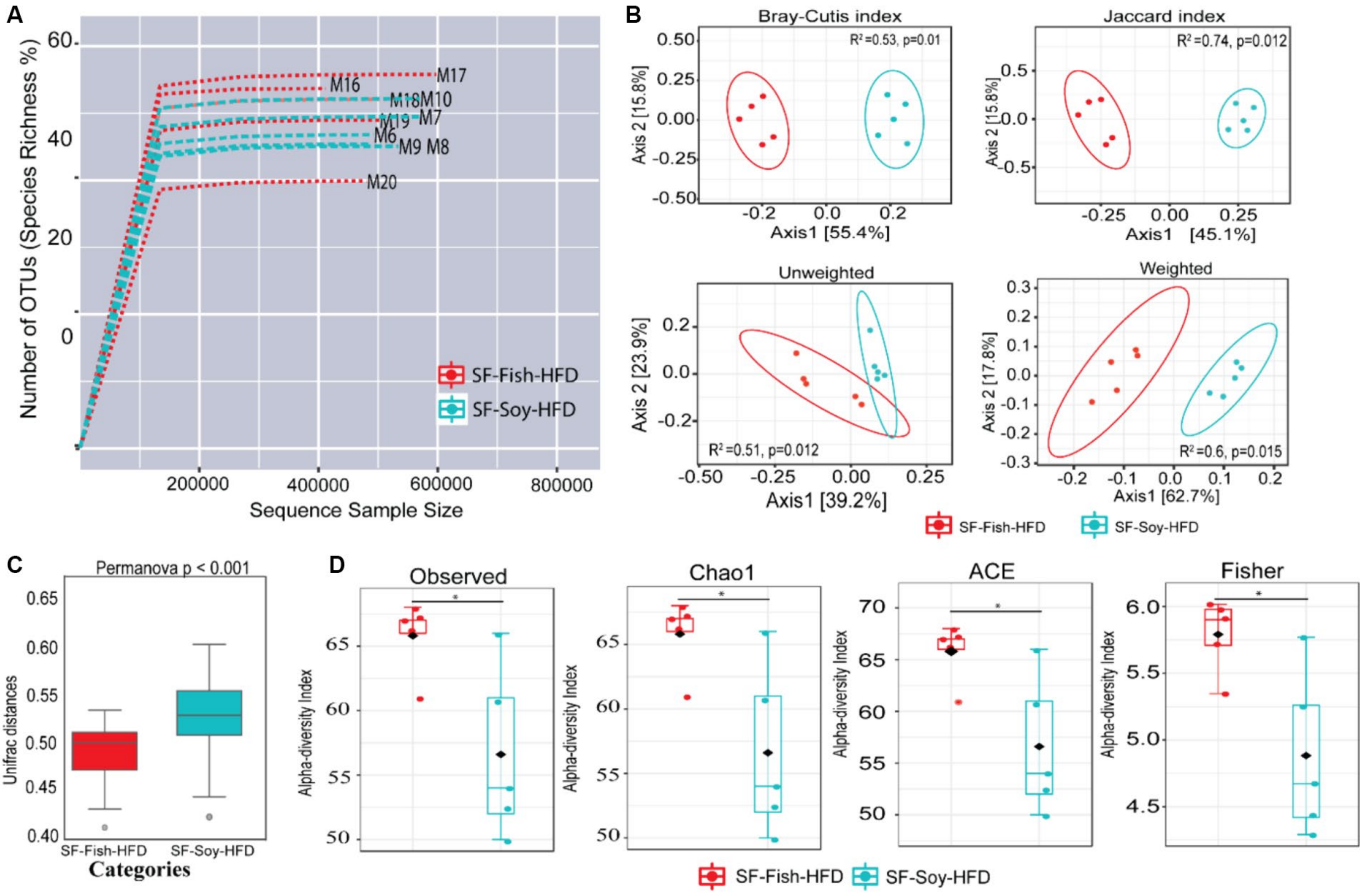
SF-Soy-HFD and SF-Fish-HFD have different effects on the gut microbiome. Analysis of gut microbiota diversity was performed between mice fed with sucrose-free soyabean oil based high fat diet (SF-Soy-HFD) and with sucrose-free fish oil based high fat diet (SF-Fish-HFD). **(A)** Influence of 16S rRNA sequencing depth on rarefaction analysis. The rarefaction curve is a plot of the number of species against the number of samples. This curve is created by randomly re-sampling the pool of N samples several times and then plotting the average number of species found for each sample. Generally, it initially grows rapidly (as the most common species are found) and then slightly flattens out (as the rarest species remain to be sampled). Rarefaction curves of different samples related to SF-Soy-HFD and SF-Fish-HFD feeding regimens are shown. **(B)** Principal component analysis (PCoA) 2D plots of beta diversity between SF-Soy-HFD and SF-Fish-HFD groups are shown. Between-sample dissimilarities were measured by using Bray-Curtis distance, Jaccard distances, unweighted and weighted UniFrac distances. **(C)** Box plots show the UniFrac distances of beta diversity between SF-Soy-HFD and SF-Fish-HFD groups. **(D)** Alpha diversity across samples from SF-Soy-HFD and SF-Fish-HFD groups is shown. Differences in alpha diversity metrics of microbial diversity and richness in the gut microbiota of mice fed with two different diets are shown as boxplots of the observed number of OTUs, Chao1, abundance-based coverage estimator (ACE) and Fisher’s alpha index. Significances marked as *p ≤ 0.05 refer to comparisons between SF-Soy-HFD and SF-Fish-HFD.

Since sucrose has an impact on microbiota dysbiosis, we speculated that a sucrose-free HFD shapes the gut microbiota, which prevents obesity in mice fed sucrose-free HFDs. Next, we focused on gut microbiome diversity, for which isolated DNA samples from the fecal contents were sequenced for the 16S rRNA V4 gene using high-throughput Illumina MiniSeq, generating 5,163,603 reads with an average of 516,360 reads per sample (Supplementary Table S3). For quality filtering, 20% of reads across all samples were cut-off, and the data were evaluated using standard quality control procedures (Schmieder and Edwards, 2011; Bolger et al., 2014). The rarefaction curve showed sequencing depth as a measure of microbial diversity, which was similar across all samples (Figure 2A). Principal component analysis (PCoA) revealed notable genetic diversity between the microbial populations in the HFDs, as measured using Bray-Curtis distance, Jaccard distances, and unweighted and weighted UniFrac distances (PERMANOVA, *p* < 0.05) (Figure 2B). UniFrac analysis further confirmed the presence of dysbiosis in SF-Soy-HFD-fed mice, compared to SF-Fish-HFD-fed mice, with a significantly greater interpersonal variation observed for the SF-Soy-HFD-fed mice (Figure 2C). Alpha diversity matrices including the observed number of OTUs, Chao1 index, ACE and Fisher’s alpha index were significantly different, each, as compared between SF-Soy-HFD and SF-Fish-HFD groups (Figure 2D).

### Differences across hierarchical taxonomy are induced by SF-Soy-HFD relative to SF-Fish-HFD feeding

**Figure 3:**
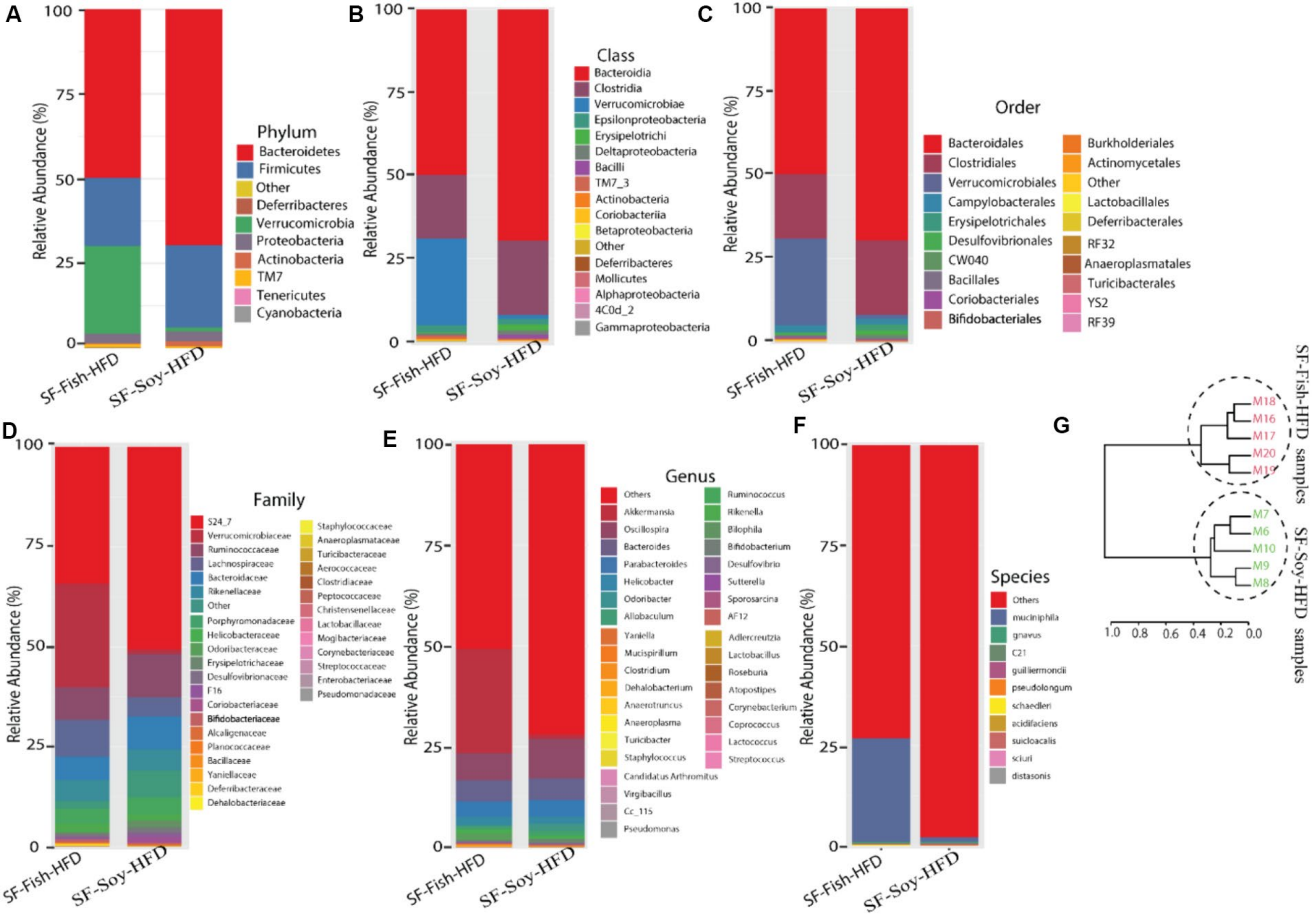
Differences across hierarchical taxonomy are induced by SF-Soy-HFD relative to SF-Fish-HFD feeding. Alterations of the composition of gut microbiota in SF-Soy-HFD and SF-Fish-HFD fed mice group. The bar plot shows taxa with average relative abundances in SF-Soy-HFD and SF-Fish-HFD at the **(A)** phylum level **(B)** Class level **(C)** order level **(D)** Family level **(E)** Genus level and **(F)** Species level. **(G)** Hierarchical clustering dendrogram of Unifrac distances of the gut microbiota for each group.

To determine the microbial distribution across the SF-Soy-HFD and SF-Fish-HFD groups, we first investigated the differential taxonomic abundance of the microbiota at different levels. The data show that the plots of relative abundances percentage of bacteria at the phylum, class, order, family, genus, and species levels were visually distinct between the SF-Soy-HFD and SF-Fish-HFD groups (Figures 3A–F; Supplementary Tables S4–S9). We observed that the relative abundances of nine bacterial taxa at the phylum level and 35 bacterial taxa at the genus level were different. At the phylum level, the nine dominant phyla were *Bacteroidetes, Firmicutes, Deferribacteres, Verrucomicrobia, Proteobacteria, Actinobacteria, TM7, Tenericutes*, and *Cyanobacteria*. Mice fed SF-Soy-HFD had a higher abundance of *Bacteroidetes, Firmicutes*, and *Deferribacteres* and a lower abundance of *Verrucomicrobia* compared to mice fed SF-Fish-HFD. The *Firmicutes*/*Bacteroidetes* (F/B) ratios in SF-Soy-HFD and SF-Fish-HFD mice were 0.34 and 0.40, respectively (Figure 3A). At the class level, *Clostridia*/*Bacteroidia* (C/B) ratios for SF-Soy-HFD and SF-Fish-HFD mice were 3.11 and 2.5, respectively (Figure 3B). At the family level, SF-Soy-HFD mice had an increased abundance of *S24-7/Muribaculaceae, Ruminococcaceae, Lachnospiraceae, Rikenellaceae* and *Acteroidaceae* and a lower abundance of *Verrucomicrobiaceae* than SF-Fish-HFD mice. Notably, the *Verrucomicrobiacae*/*S24_7* (*Muribaculaceae*) ratios for SF-Soy-HFD and SF-Fish-HFD mice were 0.02 and 0.75, respectively (Figure 3D).

At the genus level, mice fed SF-Soy-HFD showed an increased abundance of *Oscillospira, Bacteroides*, and *Parabacteroides* but a lower abundance of *Akkermansia*, as compared to mice fed SF-Fish-HFD (Figure 3E). We also determined relative abundances at the species level and found that SF-Soy-HFD mice had lower abundances of *Muciniphila, Schaedleri, Acidifaciens, Suicloacalis*, and *Distasonis* but a higher abundance of *Desulfovibrio C21-C20* and *Guilliermondii*, as compared with SF-Fish-HFD mice (Figure 3F). The hierarchical clustering dendrogram of the Unifrac distances further revealed that the gut microbial communities in mice fed SF-Soy-HFD were remarkably different from those in mice fed SF-Fish-HFD (Figure 3G).

### *S24_7/Muribaculaceae* and *Akkermansia* are the dominant taxa in SF-Soy-HFD and SF-Fish-HFD fed mice, respectively

**Figure 4:**
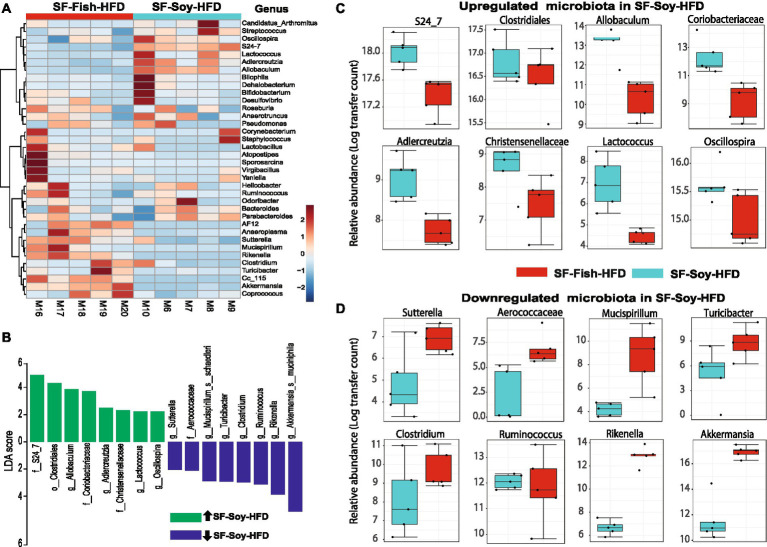
S24_7 is the dominant taxon in SF-Soy-HFD-fed mice while Akkermansia is the dominant taxon in SF-Fish-HFD-fed mice. Differences in the gut microbiome of SF-Soy-HFD-and SF-Fish-HFD-fed mice. **(A)** Hierarchically clustered heat map showing genus-level taxonomic abundance variation in the bacterial communities. Feature count abundances were scaled to allow meaningful comparison between them. The heat map was color-coded according to the intensity based on taxonomic abundances. The feature clustering pattern is presented by a dendrogram on the left side of the heat map. The correlation coefficients are represented by positive (red) or negative (blue) correlations. **(B)** Differentially abundant bacteria in SF-Soy-HFD and SF-Fish-HFD mice. Histograms of linear discriminant analysis (LDA) effect size (LEfSe) comparison between gut microbiota of SF-Soy-HFD mice (*n* = 5) and SF-Fish-HFD mice (*n* = 5). The log-level changes in the LDA score are displayed on the x-axis. Top microbiota of significance by response with an LDA score > 2 as determined using LEfSe. Orange bars: taxa found in greater relative abundance in SF-Soy-HFD group. Cyan bars: taxa found in greater relative abundance in the SF-Fish-HFD group. **(C,D)** Differential abundance of bacterial taxa in the gut microbiome of SF-Soy-HFD versus SF-Fish-HFD-fed mice.

The dominant microbial genera were identified based on the correlation coefficients of the OTUs in the SF-Soy-HFD-fed and SF-Fish-HFD-fed mice. To this end, we found that among the dominant microbial taxa, *S24_7/Muribaculaceae, Clostridiales, Allobaculum, Coriobacteriaceae, Adlercreutzia, Christensenellaceae, Lactococcus*, and *Oscillospira* were significantly positively correlated, while *Sutterella, Aerococcaceae, Mucispirillum, Turicibacter, Clostridium, Ruminococcus,* and *Rikenella* were significantly negatively correlated with the SF-Soy-HFD diet (Figure 4A).

Next, a compositional algorithm was used to search for distance patterns to identify correlations and enrichment of taxa at the genus level in relation to the SF-Soy-HFD and SF-Fish-HFD feeding interventions. This correlation was calculated based on a comparison of relative abundance using linear discriminant analysis effect size (LEfSe) (Segata et al., 2011). LDA scores >2 (*p* < 0.05) were considered significantly enriched. A total of 16 genera were changed significantly, among which eight genera were upregulated and eight genera were downregulated in SF-Soy-HFD-fed mice, compared with SF-Fish-HFD-fed mice (Figure 4B). In the SF-Soy-HFD group, the taxa that were up-regulated included *S24_7, Clostridiales, Allobaculum, Coriobacteriaceae, Adlercreutzia, Christensenellaceae, Lactococcus*, and *Oscillospira* (Figure 4C), whereas those that were down-regulated included *Sutterella, Aerococcaceae, Mucispirillum, Turicibacter, Clostridium, Ruminococcus, Rikenella*, and *Akkermansia* (Figure 4D). Overall, our results indicated a differential pattern of enrichment of gut microbial taxa in SF-Soy-HFD-and SF-Fish-HFD-fed mice.

### Functional annotation of the SF-Soy-HFD-fed mice microbiome is associated with inflammatory and metabolic disease phenotypes

**Figure 5:**
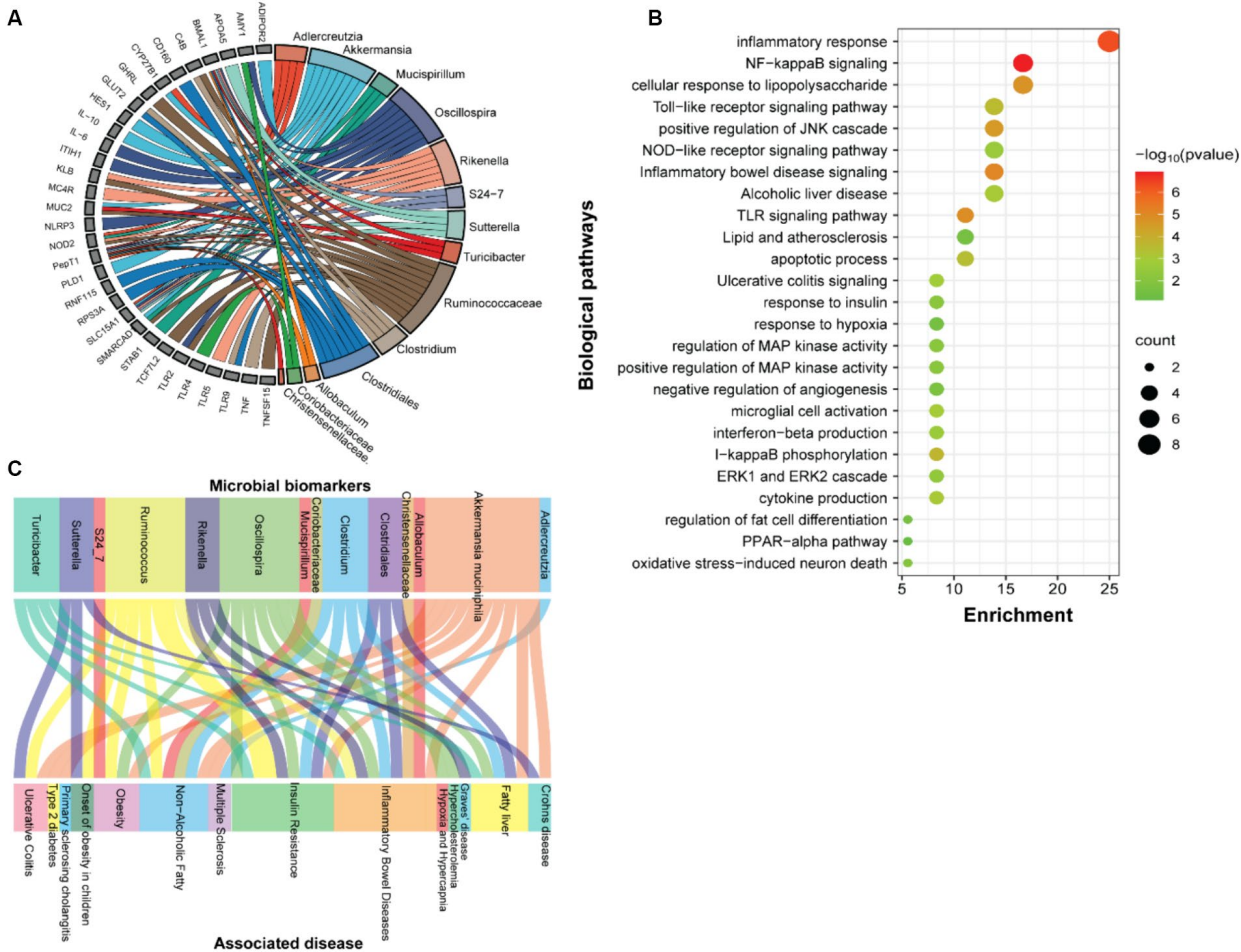
Function prediction. **(A)** Prediction of interactions between genes associated with biomarker gut microbes. **(B)** GO functional enrichment analysis of genes associated with biomarker gut microbes in SF-Soy-HFD diet versus SF-Fish-HFD diet. The functional enrichment analysis was performed using the functional annotation tool DAVID. The dot size indicates the count. The count represents the number of genes associated with each pathway. The dot color denotes the *p* values of pathways, and the x-axis represents fold enrichment. **(C)** The associations between clinical phenotypes and biomarker microbes.

We next examined the predicted association between microbiome changes and functional annotation in mice fed either SF-Soy-HFD or SF-Fish-HFD (Figure 5A). The predicted interactions between the key microbiome and host genes in SF-Soy-HFD and SF-Fish-HFD-fed mice were identified using the MIAOME database (Wang et al., 2022). Predictions from this database were derived from the associations reported in the literature. Accordingly, the taxa *Ruminococcaceae, Akkermansia, Oscillospira, Clostridiales, Rikenella, Clostridium, Adlercreutzia, Sutterella, Mucispirillum, Turicibacter*, and *S24-7/Muribaculaceae* were linked to host genes involved in previously characterized immune-metabolic pathways that mediate metabolic disorders (Figure 5A), which was further confirmed by GO functional enrichment analysis (Figure 5B).

The predicted interactions between the key microbiome and host genes in SF-Soy-HFD and SF-Fish-HFD-fed mice were identified using the MIAOME database (Wang et al., 2022). Predictions from this database were derived from the associations reported in the literature. Next, we also predicted interactions between the observed key microbiome and clinical phenotypes were identified using the MIAOME database (Wang et al., 2022). We investigated the association between microbial biomarkers and clinical phenotypes. We found that a number of inflammatory and metabolic diseases were associated with these microbial markers, including the fatty liver, NAFLD, T2D, obesity, and insulin resistance. These metabolic disorders were primarily associated with the taxa including *Ruminococcus, Akkermansia muciniphila*, *Oscillospira, Clostridium, Clostridiales*, and *Turicibacter* (Figure 5C).

### Differential metabolome shift in SF-Soy-HFD fed mice as predicted based on metabolic enzyme expression

**Figure 6:**
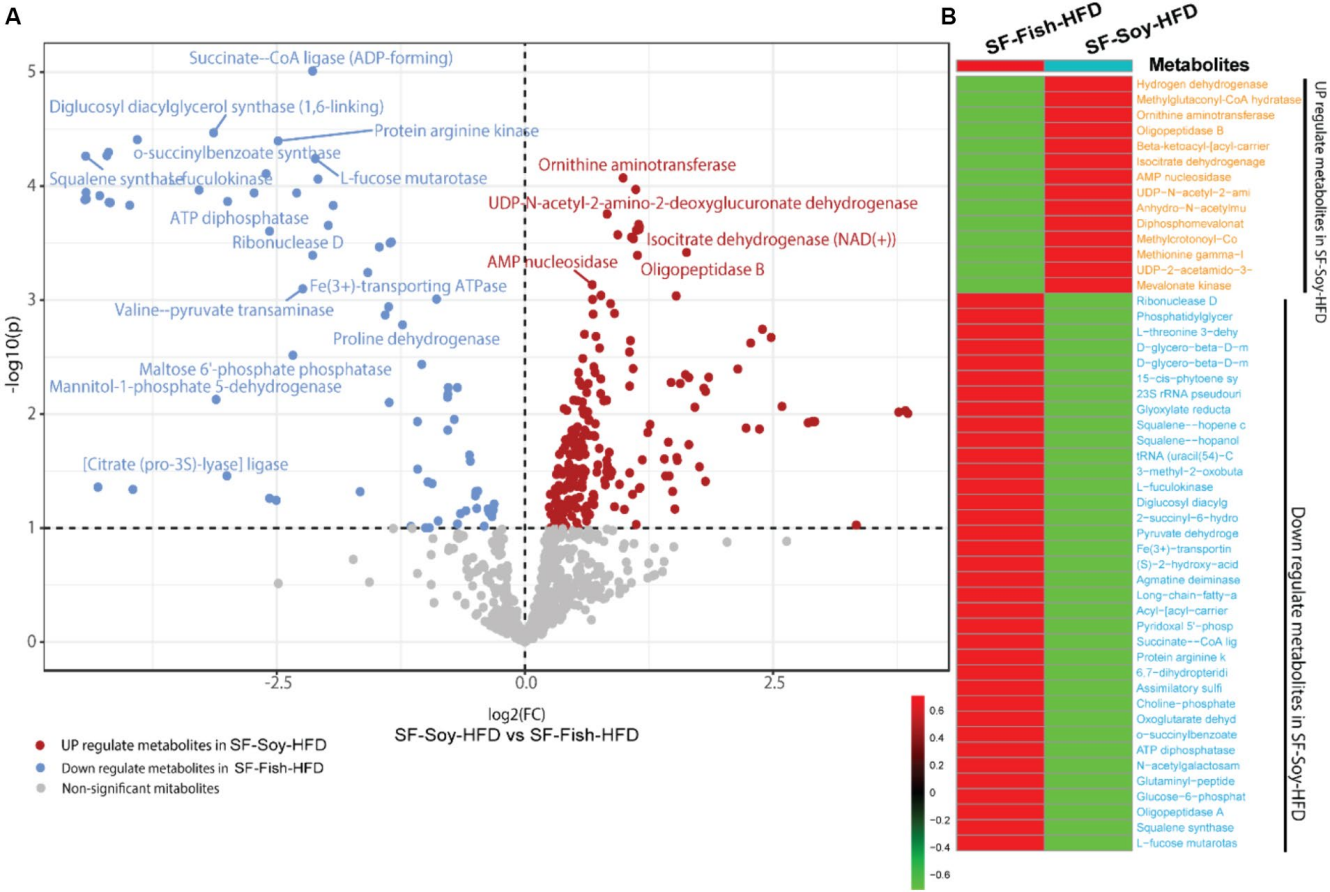
Differential microbiome upon SF-Soy-HFD feeding predicts shift in gut metabolome based on metabolic enzyme expression. Prediction of differential metabolites. **(A)** Volcano plot of differential metabolites. Volcano plot distribution highlighting the differentially expressed metabolites between SF-Soy-HFD versus and SF-Fish-HFD fed mice. The red dots denote the up-regulated and blue dots denote the down-regulated differentially expressed metabolites between SF-Soy-HFD and SF-Fish-HFD mice, whereas the brown dots denote non-significantly altered metabolites. **(B)** The heat map shows the differentially expressed metabolites between SF-Soy-HFD and SF-Fish-HFD mice.

Notably, bacterial taxa showed significant diversity between the SF-Soy-HFD and SF-Fish-HFD groups. The functional abundance of important bacterial enzymes was predicted using PICRUSt2 (Douglas et al., 2020). This analysis revealed the bacterial taxa that contributed to significant changes in the expression of bacterial enzymes associated with metabolic significance (Figure 6). Analysis of the differentially expressed genes showed that in SF-Soy-HFD mice, there was significant upregulation of ornithine aminotransferase, UDP-N-acetyl-2-amino-2-deoxyglucuronate dehydrogenase, isocitrate dehydrogenase (NAD+), AMP nucleosidase, and oligopeptidase B, and downregulation of succinate CoA ligase, diglucosyl diacylglycerol synthase (1,6 linking), protein arginine kinase, o-succinylbenzoate synthase, squalene synthase fuculokinase, L-fucose mutarotase, ATP diphosphatase, ribonuclease D, and Fe^3+^ transporting ATPase (Figure 6A). Overall, 50 metabolites showed differential abundance between the SF-Soy-HFD-and SF-Fish-HFD-fed mice. Among these, 14 were upregulated and 36 were downregulated in SF-Soy-HFD-fed mice compared to those in SF-Fish-HFD-fed mice (Figure 6B).

### Biomarker gut microbiota are associated with predicted metabolite abundance

**Figure 7:**
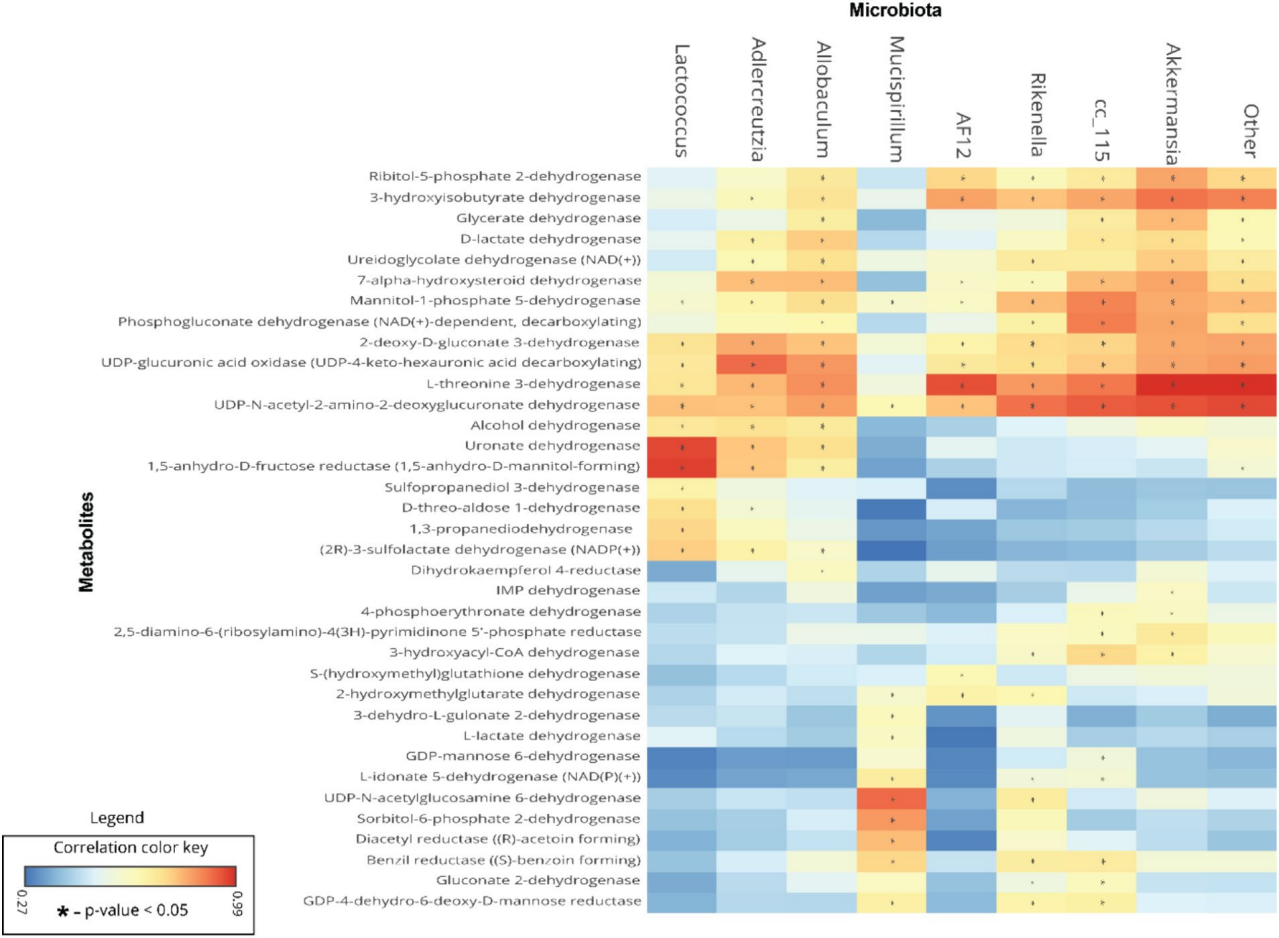
Association between the differential gut microbiome and their metabolites. To understand the association between microbial genera and metabolites, linear regression analysis was performed. A heatmap of Pearson’s correlations between the differential gut microbiome and metabolites is shown; microbial genera are displayed on *x*-axis and metabolites are shown on *y*-axis. Red and cyan colors represent a positive and a negative association, respectively. Symbols on the plot represent the level of significance with an asterisk (*), denoting Bonferroni significant associations at *p* < 0.05.

To understand the relationships between gut microbiota and the predicted metabolite profile in SF-Soy-HFD-and SF-Fish-HFD-fed mice, we used Pearson’s correlation analysis of the differential gut microbiome and metabolites using microbiome analyst (Lamichhane et al., 2021). Correlation analysis revealed a positive correlation between the gut microbiota (*Lactococcus, Adlercreutzia, Allobaculum, g_AF12 (Rikenellaceae family), g_Cc_115 (Erysipelotrichaceae family), Rikenella* and *Akkermansia*) and bacterial enzymes. The generated heatmap showed that *Akkermansia, g_AF12 (Rikenellaceae family), g_Cc_115 (Erysipelotrichaceae family), Rikenella, Allobaculum, Adlercreutzia, Lactococcus* and other OTUs had a highly significant positive association with a wide spectrum of metabolites including 3-hyroxysobutyrate dehydrogenase, UDP-4-keto hexauronic acid decarboxylation, L-threonine 3-dehydrogenase, UDP-N-acetyl-2-amino-2-deoxyglucuronate dehydrogenase, uronate dehydrogenase, and alcohol dehydrogenase. Alternatively, *Lactococcus, Allobaculum, Adlercreutzia, g_AF12 (Rikenellaceae family)* and *Akkermansia* were negatively correlated with a range of other bacterial metabolites, including 3-hydroxyacyl-CoA, dehydrogenase, 2-hydroxymethylglutarate dehydrogenase, S-(hydroxymethyl) glutathione dehydrogenase, L-lactate, dehydrogenase, L-idonate 5-dehydrogenase, (NAD(P)(+))UDP-N-acetylglucosamine 6-dehydrogenase, GDP-mannose 6-dehydrogenase, and 3-dehydro-L-gulonate 2-dehydrogenase (Figure 7).

### Liver steatosis and inflammation in mice fed with SF-Soy-HFD

**Figure 8:**
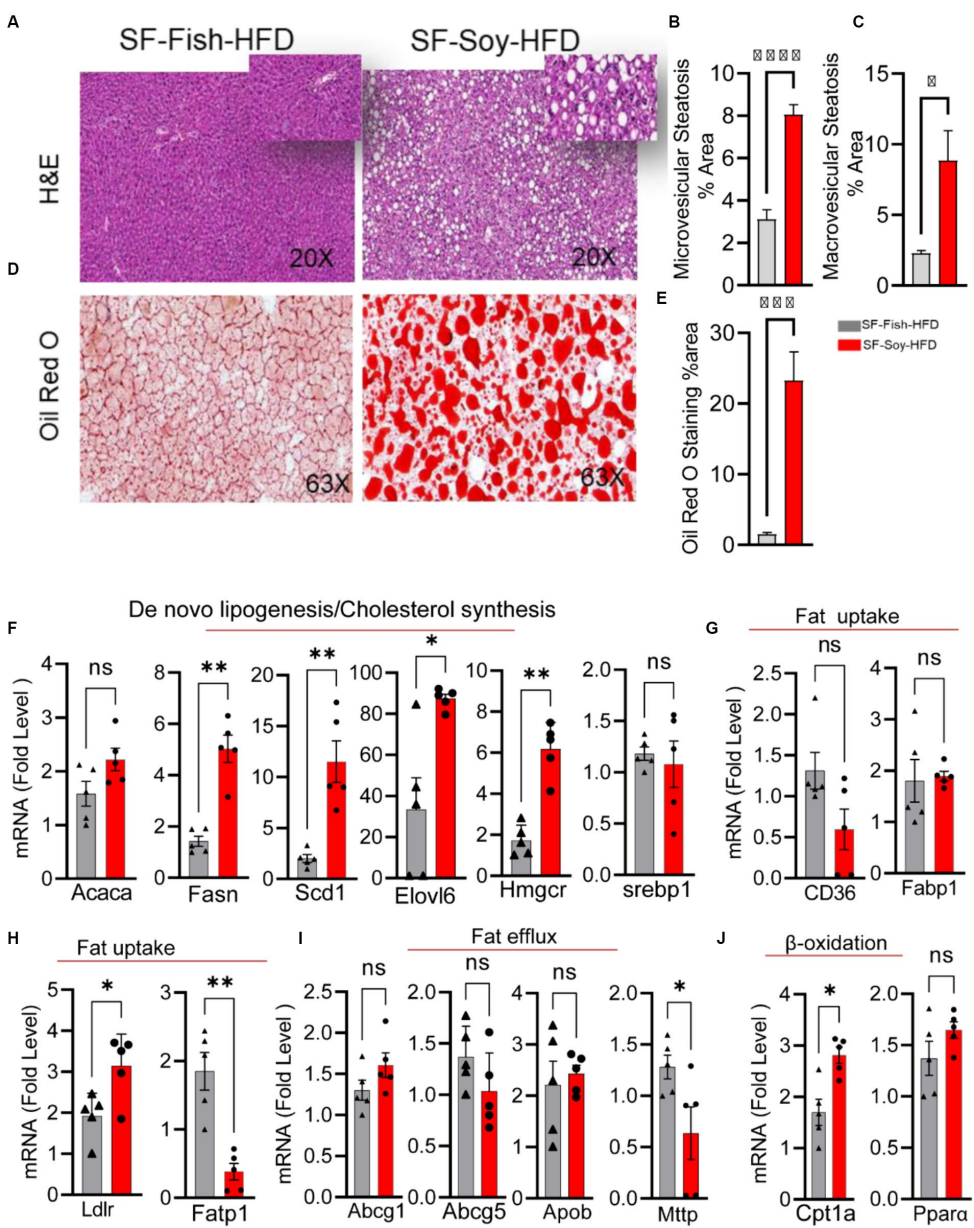
SF-Soy-HFD diet leads to hepatic steatosis and induction of entire lipogenic program. **(A)** Livers from mice (*n* = 5 per group) fed 24 weeks SF-Fish-HFD or SF-Soy-HFD were sectioned and stained with H&E (original magnification, ×40). **(B,C)** The percentage of microvescicular and macrovescicular was assessed. **(D,E)** Lipid accumulation displayed by Oil Red O staining of the liver sections from mice fed for 20 weeks with either SF-Fish-HFD or SF-Soy-HFD. % area of Oil Red O staining is shown. **(F)** mRNA expression of genes related to *de novo* lipogenesis (*Acaca, Fasn, Scd1, Elovl6*) and cholesterol synthesis (*Hmgcr, Srebp1*). **(G,H)** mRNA expression of genes related to fat uptake (*CD36, Fabp1, Ldlr, Fatp1*). **(I)** mRNA expression of genes related to fat efflux (*Abcg1, Abcg5, Apob, Mttp*). **(J)** mRNA expression of genes related to β-oxidation (*Cpt1a, Ppara*). Data are presented as mean ± SEM. Significance was determined by using unpaired Student’s *t*-test. *p < 0.05 for all data.

We speculated that the pathological shift in the gut microbiota of mice fed SF-Soy-HFD could influence liver function and/or peripheral insulin sensitivity. As shown in Figures 8A–C, the livers from SF-Soy-HFD-fed mice displayed mixed steatosis of microvesicular and macrovesicular types. No signs of fat accumulation were detected in livers isolated from mice fed the SF-Fish-HFD (Figures 8A–C). These gross changes were further confirmed using Oil Red O staining of liver sections isolated from both mouse groups. The animals fed SF-Soy-HFD showed notable steatosis with high fat accumulation, as compared to the livers from SF-Fish-HFD fed mice (Figures 8D,E).

Consistent with the development of steatosis, gene expression analysis of liver tissues isolated from mice fed SF-Soy-HFD showed a statistically significant upregulation of genes involved in: (i) *de novo* lipogenesis (Fatty acid synthase: *Fasn*), and (Stearoyl-coenzyme A desaturase 1: *Scd1*), (ii) Triglyceride (TG) synthesis (elongation of very long-chain fatty acid-like fatty acid elongase 6: *Elovl6*), and (iii) cholesterol synthesis (HMG-CoA reductase: *Hmgcr*) pathways, compared with expression in liver samples from SF-Fish-HFD fed mice, whereas no significant difference was found in the expression of acetyl-CoA carboxylase alpha (*Acaca*) and sterol regulatory element-binding protein 1 (*Srebp1*) between the two groups (Figure 8F). Regarding fat uptake, low density lipoprotein receptor (*Ldlr*) expression was significantly upregulated and long-chain fatty acid transport protein 1 (*Fatp*) expression was downregulated in the liver tissues of SF-Soy-HFD-fed mice compared with those from SF-Fish-HFD-fed mice. However, no significant difference was observed in the expression of fatty acid transporter *Cd36* and fatty acid binding protein 1 (*Fabp1*) between the two groups (Figures 8G,H). Regarding fat efflux, microsomal triglyceride transfer protein (*Mttp*) expression was significantly downregulated in SF-Soy-HFD-fed mice compared with SF-Fish-HFD-fed mice, but no significant difference was observed in the expression of ATP-binding cassette sub-family G member (*Abcg1*)-*1, Abcg5*, and apolipoprotein B (*Apob*) between the two groups (Figure 8I). In relation to the expression of β-oxidation genes, carnitine palmitoyltransferase 1A (*Cpt1a*) transcripts were significantly upregulated, while peroxisome proliferator-activated receptor α (*Ppara*) transcripts were slightly increased in the liver tissues from SF-Soy-HFD fed mice compared with SF-Fish-HFD fed mice (Figure 8J).

**Figure 9:**
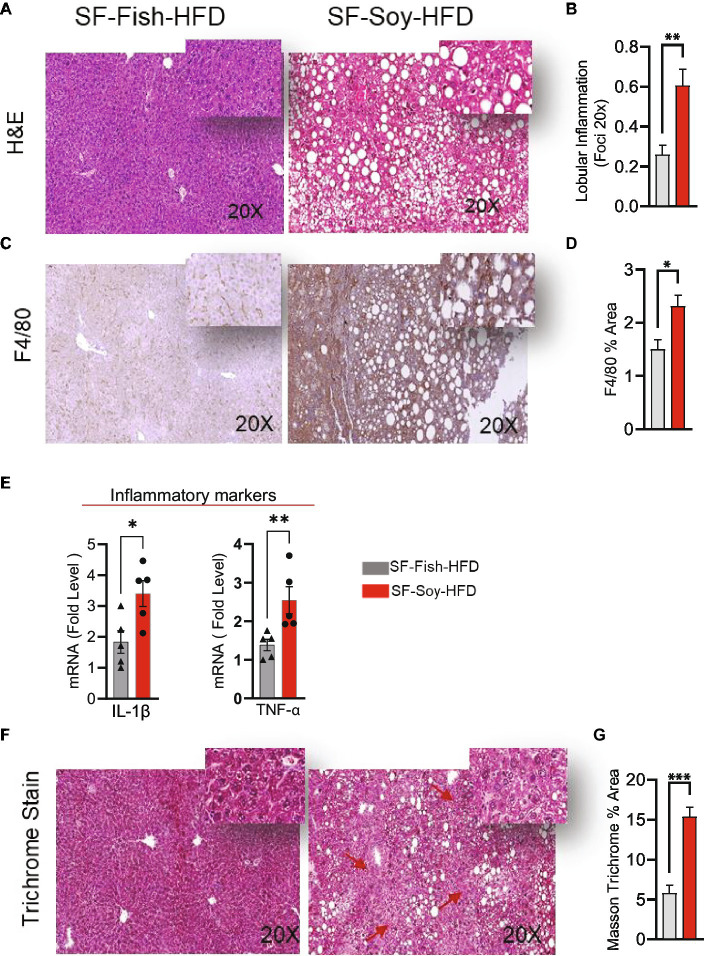
SF-Soy-HFD leads to hepatic inflammation. **(A,B)** Representative images of hematoxylin and eosin (H&E) staining of the liver sections of mice fed 24 weeks SF-Soy-HFD or SF-Fish-HFD. Lobular inflammation was assessed. **(C)** Hepatic infiltrates were stained by immunohistochemistry for macrophage (F4/80) markers. **(D)** F4/80% area. **(E)** mRNA expression of genes related to inflammation (IL-1β, TNF-α) was determined by real-time RT-qPCR. **(F,G)** Comparison of collagen deposition as assessed by Masson’s trichrome staining in the liver sections. Data are presented as mean ± SEM. Significance was determined by using unpaired Student’s *t*-test. *p < 0.05 for all data.

We next assessed inflammation in liver tissues isolated from mice fed SF-Fish-HFD or SF-Soy-HFD. Unlike samples from SF-Fish-HFD-fed mice, liver histopathology of mice fed with SF-Soy-HFD samples revealed lobular inflammation (Figures 9A,B). Furthermore, SF-Soy-HFD feeding led to the accumulation of F4/80-positive macrophages in the liver tissues, suggesting inflammation (Figures 9C,D). Consistent with macrophage infiltration and lobular inflammation, gene expression of interleukin 1 beta (IL-1β) and tumor necrosis factor alpha (TNF-α) was significantly upregulated in the liver samples from SF-Soy-HFD-fed mice compared with those from SF-Fish-HFD-fed mice (Figure 9E).

Generally, the liver tissues collected from SF-Soy-HFD-fed mice exhibited abnormal morphologies with atypical yellowish discoloration, while no such gross changes were observed in the livers of SF-Fish-HFD-fed mice. In addition, in liver sections from SF-Soy-HFD fed mice, notable fibrosis was observed, following trichrome staining (Figure 9F).

### Mice fed with SF-Soy-HFD displayed impaired glucose tolerance, insulin resistance, and lower circulating leptin levels

**Figure 10:**
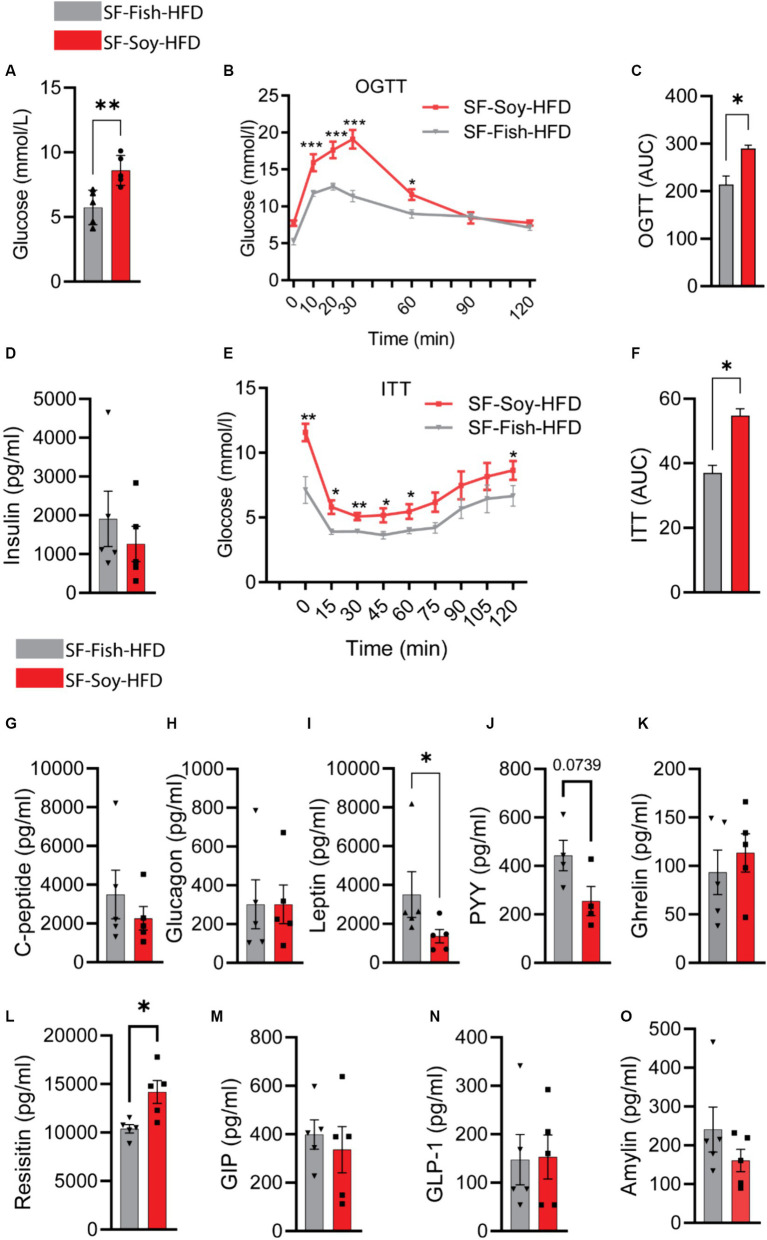
Mice fed with SF-Soy-HFD displayed impaired glucose tolerance, insulin resistance, and lower circulating leptin levels. **(A)** Fasting blood glucose was measured at 21 weeks of dietary intervention. **(B,C)** Oral glucose tolerance test was performed after 21 weeks and area under the glucose concentration curve (AUC). **(D)** Fasting plasma insulin level was measured. **(E,F)** Insulin tolerance test after 22 weeks of HFD and area under the glucose concentration curve (AUC). **(G–O)** Fasting plasma metabolic markers levels were determined using Milliplex assay kits. Data are presented as mean ± SEM.

Clinical analyses were performed to further investigate the potential implications of gut dysbiosis in SF-Soy-HFD-fed mice regarding metabolic impairment. Initially, we observed an increase in the fasting blood glucose levels in plasma samples from SF-Soy-HFD-fed mice compared to those from SF-Fish-HFD-fed mice (Figure 10A). Furthermore, OGTT analysis revealed a significant reduction in glucose tolerance in SF-Soy-HFD-fed mice (Figure 10B), and the area under the curve (AUC) was significantly higher in this group than in the SF-Fish-HFD group (Figure 10C).

Although the fasting plasma insulin levels did not differ significantly between the two mice groups (Figure 10D), ITT analysis showed poor plasma insulin levels in SF-Soy-HFD-fed mice compared with SF-Fish-HFD-fed mice, indicating the induction of insulin resistance in this dietary intervention group (Figures 10E,F).

Next, we compared plasma metabolic hormone levels and, as shown in Figures 10G–O, the levels of C-peptide, glucagon, ghrelin, gastric inhibitory polypeptide (GIP), glucagon-like peptide 1 (GLP-1), and amylin were comparable in both groups. In SF-Soy-HFD-fed mice, leptin and Peptide YY (PYY) levels were reduced, whereas resistin levels were elevated, compared with SF-Fish-HFD-fed mice.

## Discussion

Almost all HFDs promote weight gain, cause hepatic steatosis, and increase inflammatory cytokines in the liver (Echeverría et al., 2019). These effects are due to the high fat and sucrose contents of the diet. Interestingly, different dietary fats have unequal contributions to obesity and the gut microbiome landscape (Caesar et al., 2015, 2016; Kübeck et al., 2016; Li et al., 2017; Portune et al., 2017; Prieto et al., 2018), which may be due to the different contents of saturated fatty acids (SFA) and unsaturated fatty acids (UFA) (An et al., 2022). Fish oil and soybean oil are considered healthy dietary fats; however, these oils have a distinct impact on the gut microbiome (Li et al., 2017). To assess these fats based on their properties alone, without the influence of sucrose, we used dietary models of sucrose-free (SF) Soy-HFD and SF-Fish-HFD-feeding. Using these models, we analyzed gut microbiome shifts and their impact on liver steatosis, inflammation, hepatic fibrosis, systemic glucose tolerance, and insulin resistance.

In our study, UniFrac analysis revealed a clear separation between microbial communities of Soy-HFD and Fish-HFD groups in a PCoA plot, indicating significant differences in the microbial composition of two gut microbiomes. It implies that the Soy-HFD diet feeding had altered the gut microbial landscape. A significantly greater interpersonal variation observed for Soy-HFD group, compared with Fish-HFD, was represented by larger spread or clustering in the PCoA plot which suggests that the Soy-HFD feeding leads to more diverse and individualized microbial responses. Notably, this increased variation is considered a hallmark of gut dysbiosis, indicating a disrupted and less stable gut microbiome comprising the phylogenetically distinct microbial communities. Together, these data provide insight into how Soy-HFD feeding in mice might impact the composition and stability of the gut microbiome and thereby affect the host health.

Our data showed that SF-Soy-HFD feeding resulted in a different microbiome landscape shift than that induced by SF-Fish-HFD feeding. The SF-Soy-HFD group had an increased abundance of *Firmicutes, Bacteroidetes,* and *Deferribacteres* and a lower abundance of *Verrucomicrobia* than the SF-Fish-HFD group. The F/B ratio was relatively higher in the SF-Fish-HFD group than in the SF-Soy-HFD group. The significance of the F/B ratio as a predictive biomarker of disease remains controversial, as some studies found that the gut microbiota of individuals with obesity had a higher F/B ratio (Ley et al., 2005), while others did not observe any remarkable alterations in the F/B ratio between normal-weight and obese individuals (Karlsson et al., 2012; Hu et al., 2015). Metabolically, *Firmicutes* are more effective than Bacteroidetes in deriving energy from food, thus promoting higher absorption of calories and subsequent weight gain. However, we did not observe significant differences in body weight gains between SF-Soy-HFD-and SF-Fish-HFD-fed mice, which may be because only a slight difference was observed in the F/B ratio (0.34 versus 0.40). Another factor that might account for this discrepancy is that sucrose-free HFDs were fed to mice, whereas increased sucrose consumption primed mice for enhanced caloric intake, hyperphagic behavior, and weight gain during HFD feeding (Togo et al., 2019).

At the class level, the C/B ratio was higher in the SF-soy-HFD group (3.11) than in the SF-fish-HFD group (2.5). The balance between gut microbiota, such as *Clostridia* and *Bacteroidia*, is critical for maintaining gut health. *Bacteroidia* are the predominant colonizers of the colon but are also present in other parts of the gut, such as oral cavity, throat, and stomach, and these microbiota can account for nearly 50% of the 16S rRNA sequences detected in healthy human mucosal tissue samples (Eckburg et al., 2005). Functionally, their interactions with the host are more mutualistic than commensalism. They play a key role in fermentation process and biosynthesis of short-chain fatty acids (SCFAs), including butyric, acetic, and propionic acids, and provide >50% of the total caloric supply. SCFAs have anti-inflammatory and antineoplastic properties, and support gut health (Ríos-Covián et al., 2016). *Bacteroidia* interact with the gut immune system, activate T-cells, regulate bile acid homeostasis, and prevent gut colonization by pathogenic bacteria via competitive inhibition (Mazmanian et al., 2008). In contrast, expansions in *Clostridia* are linked to immune-metabolic impairment, and such gut dysbiosis may lead to disruptions in gut permeability, chronic inflammation, increased susceptibility to infections or certain immune disorders, and gut metabolic changes associated with obesity, T2D, and NAFLD (Kiseleva et al., 2023).

Our correlational analysis between microbial biomarkers and associated clinical phenotypes revealed that microbiome changes in *Ruminococcus, Akkermansia muciniphila*, *Oscillospira, Clostridium, Clostridiales*, and *Turicibacter* were related to metabolic disorders including obesity, T2D, insulin resistance, fatty liver, and NAFLD. Briefly put, the abundance of *Ruminococci* (including *Oscillospira*) in the gut microbiome is affected in obesity which alters glucose/energy metabolism as well as lipid metabolism and fat storage (Moreno-Navarrete et al., 2018), further contributing to metabolic inflammation, insulin resistance, T2D, and hepatic steatosis. Since *Ruminococci* and *Oscillospira* are involved in SCFAs production, such as butyrate, their dysbiosis can lead to increased inflammation – a common underlying factor in several metabolic disorders (Baxter et al., 2019; Yang et al., 2021). *Akkermansia muciniphila* is a mucin-degrading bacterium in the gut and it may have both beneficial and detrimental effects, depending on its abundance and health of the host. Given its key roles in maintenance of gut barrier integrity and anti-inflammatory responses via the production of SCFAs (Xu et al., 2020; Rodrigues et al., 2022), a lower abundance of *A. muciniphila* has been associated with obesity, T2D, metabolic syndrome, and NAFLD (Niu et al., 2024). Similarly, Clostridial dysbiosis has been linked to changes in glucose/energy, and lipid metabolism, contributing to obesity/T2D, insulin resistance, and NAFLD, depending on the species involved and the overall composition of the gut microbiota (Jin and Xu, 2023). *Turibacter* is involved in the maintenance of gut barrier integrity, immune modulation, energy regulation, and production of SCFA butyrate, therefore, its dysbiosis is often associated with metabolic disorders including obesity (Acharya et al., 2019).

Interestingly, at the family level, SF-Fish-HFD-fed mice showed a higher abundance of *Verrucomicrobiaceae* and a lower abundance of Lachnospiraceae and Rikenellaceae than SF-Soy-HFD mice. In agreement with these findings, Cui et al. found that the improvement in gut microbiota composition by dietary intervention led to an increased abundance of beneficial *Verrucomicrobia* and a reduced abundance of *Lachnospiraceae* and *Actinomycetaceae* (Cui et al., 2022). Similarly, at the genus level, SF-Soy-HFD feeding led to a lower abundance of *Akkermansia* than SF-Fish-HFD, which implies that soy-based HFD feeding in mice promotes dysbiosis with a negative impact on the beneficial *Akkermansia* which is consistent with our previous finding that SF-Soy-HFD favors lower abundance of *Verrucomicrobiaceae* family members. A cross-sectional study found a negative association between *Akkermansia* in *Verrucomicrobiaceae* and metabolic syndromes, including obesity, diabetes, and cardiovascular disease (Zhou et al., 2021). Furthermore, the altered microbiota representing gut dysbiosis in the SF-Soy-HFD group was predicted to be associated with key disease pathways, including inflammation, NF-κB and JNK signaling, lipopolysaccharide responses, Toll-like receptor signaling, NOD-like receptor signaling, inflammatory bowel disease, liver disease, atherosclerosis, and apoptosis. Although HFDs are known to promote the development of diseases via alterations in the gut microbiome (Cani, 2018; Durack and Lynch, 2019), our study adds that, even in the absence of sucrose, a soybean-based HFD can have a detrimental effect on gut health and induce dysbiosis. Interestingly, fish oil-based sucrose-free HFD feeding did not induce gut dysbiosis in mice. Previous work also showed that fish oil has a different effect on the gut microbiome than soybean oil, which corroborates our findings. In contrast, Li et al. reported that fish oil induces the expression of more inflammatory factors than soybean oil (Li et al., 2017). However, we did not find such an effect with SF-F-HFD, which could have been due to the lack of sucrose in the diet.

Moreover, analysis of the predicted metabolomic profiling of microbiota communities in the SF-Soy-HFD and SF-Fish-HFD groups revealed that of the 50 metabolites that were differentially expressed between the two dietary groups, 14 metabolites were upregulated and 36 metabolites were downregulated in the SF-Soy-HFD group, compared with the SF-Fish-HFD group. Indeed, bacterial metabolites may vary depending on the type of substrate the microbiota is exposed to, most likely due to the different nutrient compositions of these two dietary fats. Soybean oil has two main polyunsaturated fatty acids, linoleic acid (omega-6) and alpha-linolenic acid (omega-3), whereas fish oil is rich in long-chain omega-3 fatty acids such as eicosapentaenoic acid and docosahexaenoic acid (Li et al., 2017). The bioavailability of different types and amounts of these fatty acids may influence the production of SCFAs, bacterial enzymes, and metabolites by gut microbiota. Microbial communities and metabolites can be affected by secondary bile acids produced in the gut and play a role in signaling between microbiota and host cells. By regulating local inflammatory responses, resolvins and eicosanoids produced variably by n-6 fatty acid-rich soybean oil and n-3 fatty acid-rich fish oil may also affect gut microbial composition and metabolites (Bosco et al., 2013). In addition, phytochemicals, such as isoflavones found in soybean oil (Soyata et al., 2021) and astaxanthin, an antioxidant found in fish oil (Saw et al., 2013) contribute differentially to gut microbial taxa and metabolites.

We speculate that the peculiar gut microbiome shift in SF-Soy-HFD-fed mice might be linked to liver pathology and changes in insulin sensitivity, as the livers from these mice displayed increased fat accumulation, indicating steatosis; consistent with this, *de novo* lipogenesis and TG synthesis genes were found to be upregulated in liver samples of these mice. These findings are in line with previous evidence that soybean oil induces excessive hepatic lipid accumulation and impairs glucose metabolism in fish (Li et al., 2016; Tan et al., 2023). We believe that a high proportion of C18:2 n-6 polyunsaturated fatty acids in soybean oil could favor lipid accumulation in the liver, given that polyunsaturated fatty acids have been reported as secondary targets for β-oxidation, after the primary targets that include monounsaturated and saturated fatty acids (Bell et al., 2001).

Additionally, expression of the genes associated with cholesterol synthesis (*Hmgcr, Srebp1*), β-oxidation (*Cpt1a* and *Pparα*), and low-density lipoprotein receptor (*Ldlr*) was increased in mice fed SF-Soy-HFD, while there was no significant upregulation of the fat uptake genes (*Cd36, Fabp1*). In contrast, the fatty acid transport protein 1 (*Fatp1*) and fat efflux (*Mttp*) genes were downregulated. Overall, these changes suggest that SF-Soy-HFD feeding in mice might lead to steatosis by favoring *de novo* lipogenesis over β-oxidation and reducing fatty acid transport and efflux. Arguably, soybean oil is rich in polyunsaturated fats (~55% n-6 fatty acids), and the liver and plasma metabolomics in mice identified a positive correlation between obesity and hepatic C18 oxylipin, a metabolite of n-6 fatty acid linoleic acid (C18:2) (Deol et al., 2017). Emerging evidence suggests that diets rich in soybean oil are associated with increased adiposity, diabetes, insulin resistance, and fatty liver (da Costa et al., 2011; Deol et al., 2015). Not surprisingly, Deol et al. showed that soybean oil induced more metabolic effects in mice than an isocaloric diet mostly comprised of saturated fats and containing fructose and coconut oil (Deol et al., 2015). Another study showed that soybean oil consumption led to elevated serum cholesterol, metabolic syndrome comorbidities, and NAFLD (Morrison et al., 2015). These studies are consistent with our findings of steatosis in mice fed SF-Soy-HFD.

Consistent with the positive association between gut microbial dysbiosis and inflammatory pathways, the livers from mice fed SF-Soy-HFD, but not SF-Fish-HFD, showed lobular inflammation, fibrosis, and accumulation of macrophages. Genes related to hepatic inflammation (*Il1b* and *Tnfa*) were upregulated in the livers of SF-Soy-HFD fed mice compared to those in mice fed SF-Fish-HFD. Regarding the proinflammatory effects of SF-Soy-HFD feeding in mice, it is notable that arachidonic acid (C20:4), a long-chain ω-6 fatty acid found predominantly in hepatic phospholipids, induced proinflammatory responses in the liver and impaired hepatic and adipose functions, whereas ω-3 fatty acids did not have such effects (Scorletti and Byrne, 2013). Indeed, individuals with NAFLD have a higher hepatic phospholipid n-6 to n-3 polyunsaturated fatty acid (PUFA) ratio than healthy individuals (Elizondo et al., 2007). Nonetheless, others have shown that reducing the dietary n-6 to n-3 PUFA ratio by α-linolenic acid (C18:3) supplementation did not attenuate HFD-induced obesity or associated metabolic and inflammatory outcomes (Enos et al., 2014).

Omega-6 based diets, such as soybean oil, can lead to a potential microbiome shift and metabolic endotoxemia due to a relative increase in LPS-producing *Enterobactericeae* family members or a relative decrease in LPS-suppressing *Bifidobacterium*, with changes in intestinal permeability (de La Serre et al., 2010; Moreira et al., 2012). In our study, SF-soy-HFD feeding in mice supported the abundance of *Bacteroidetes, Firmicutes,* and *Deferribacteres* and reduced the abundance of *Verrucomicrobia*. Notably, the reduced abundance of *Verrucomicrobia* is in agreement with increased inflammatory responses in these mice, as some *Verrucomicrobia* act as mucin metabolizers and maintain gut barrier integrity, play key roles in reducing LPS-induced inflammation, and in maintaining insulin sensitivity and metabolic health (Chelakkot et al., 2018; Raftar et al., 2022). Linoleic acid, an ω-6 fatty acid found in high concentrations in soybean oil, can promote dysbiosis and inflammation. Arachidonic acid, another ω-6 fatty acid, was found to increase gut permeability for absorption of bacterial endotoxins, leading to the induction of inflammatory cytokines, including TNF-α and IL-1β (Tsalamandris et al., 2019), which is consistent with the increased expression of these cytokines in the livers of mice that were fed with SF-Soy-HFD. Some discrepancies found in previous studies might account for the confounding factors related to the effects of animal age, genetics, dietary composition, and feeding regimens on gut microbial diversity and functions.

This study involves several important implications as it highlights that the type of fat source (Soybean oil vs. Fish oil) in the diet may lead to different outcomes regarding changes in gut microbiome and associated metabolic disorders. Based on our findings, soy-based fats might be more detrimental to the liver health, compared to fish-based fats. Our study underscores the importance of considering specific dietary components as opposed to only the macronutrient content. Notably, individual responses to dietary fats can vary and, therefore, personalized diets can be tailored for humans based on genetic backgrounds, specific health conditions, and the gut microbiome profiles. Our study also points to the critical role of gut-liver axis, whereby the gut dysbiosis induced by Soy-HFD feeding might lead to changes in gut permeability, liver inflammation and steatosis. More importantly, these findings have a potential for human health translation and inform dietary guidelines and recommendations.

Nonetheless, this study remains limited by certain caveats. First, additional low fat controls were not included. Second, metabolomic analysis of serum samples was not performed to study the systemic profiles of absorbed gut bacterial metabolites. Third, it remains to be seen whether the same type of gut dysbiosis and/or inflammatory changes are induced by the intake of similar diets in humans. It is evident that these aspects remain unexplored; therefore, future investigations should be directed to address these concerns, aiming to provide a clearer understanding of the gut dysbiosis induced by soybean-based HFDs in both animals and humans.

In conclusion, our data support that, as opposed to SF-Fish-HFD, SF-Soy-HFD feeding in mice promotes the gut dysbiosis that concurs with liver steatosis and inflammation, implying that intake of soybean-based HFD may have deleterious consequences for metabolic health.

## Data availability statement

The datasets presented in this study can be found in online repositories. The names of the repository/repositories and accession number(s) can be found at: https://www.ncbi.nlm.nih.gov/, PRJNA1013708.

## Ethics statement

The animal study was approved by Dasman Diabetes Institute Institutional Animal Care and Use Committee (RA AM-2016-007). The study was conducted in accordance with the local legislation and institutional requirements.

## Author contributions

TJ: Data curation, Formal analysis, Writing – original draft, Writing – review & editing, Methodology. SaS: Formal analysis, Writing – original draft, Writing – review & editing, Conceptualization, Methodology. AH: Formal analysis, Writing – original draft, Writing – review & editing. MM: Data curation, Formal analysis, Investigation, Methodology, Software, Validation, Visualization, Writing – original draft, Writing – review & editing, Conceptualization. HA: Formal analysis, Writing – original draft, Writing – review & editing, Data curation, Methodology. FA-R: Data curation, Formal analysis, Writing – review & editing. RN: Data curation, Writing – review & editing, Formal analysis, Methodology. SK: Data curation, Formal analysis, Writing – original draft, Writing – review & editing, Investigation, Methodology. RT: Writing – review & editing, Data curation, Formal analysis, Methodology. FB: Writing – review & editing, Data curation, Formal analysis. StS: Writing – review & editing, Data curation, Formal analysis, Methodology. AW: Writing – review & editing, Data curation, Formal analysis. NaA: Writing – review & editing, Data curation, Formal analysis. AA-R: Writing – review & editing, Data curation, Formal analysis. NeA: Writing – review & editing, Data curation, Resources. SA: Writing – review & editing, Data curation, Formal analysis. MA-F: Writing – review & editing, Data curation, Formal analysis. AA: Writing – review & editing, Data curation, Formal analysis, Resources. FA: Formal analysis, Writing – review & editing, Data curation. TT: Formal analysis, Writing – review & editing, Software. HK: Formal analysis, Writing – review & editing. JT: Formal analysis, Writing – review & editing, Investigation. FA-M: Formal analysis, Writing – review & editing, Conceptualization, Data curation, Resources, Software. RA: Conceptualization, Formal analysis, Funding acquisition, Investigation, Project administration, Resources, Supervision, Validation, Writing – original draft, Writing – review & editing.
